# ASO Author Reflections: Achieving Surgical De-Escalation of Breast Cancer Through Cryoablation

**DOI:** 10.1245/s10434-023-14335-0

**Published:** 2023-09-27

**Authors:** Michael W. Melkus, Sonia Y. Khan, Jaclyn Cole, Rakhshanda Layeequr Rahman

**Affiliations:** Breast Center of Excellence and Department of Surgery, School of Medicine, Texas Tech University Health Sciences Center, Lubbock, TX USA

## Past

Since the 1970s, there has been a paradigm shift in the understanding of breast cancer from local tumor burden that requires aggressive local surgical therapy to a systemic disease from the outset. This paved the way toward de-escalation from mastectomy to breast-conservation surgery, and from axillary dissection to sentinel node biopsy over the last 5 decades. This was achieved primarily due to an earlier diagnosis resulting from better screening techniques. Additionally, targeted adjunctive therapies and genomic profiling of cancers that can accurately identify tumors that do not require toxic therapies has also de-escalated the use of systemic therapies. Approaches to improve surgical de-escalation continue to evolve, with less invasive strategies such as percutaneous tumor destruction with cryoablation.

## Present

Breast cancer cryoablation has primarily concentrated on low-risk small tumors for both the elderly and poor surgical candidates. The American College of Surgeons Oncology Group (ACOSOG) 1072 study was the first large breast cancer cryoablation clinical study focused on unifocal invasive ductal carcinoma ≤2 cm, with subsequent surgical resection, that demonstrated successful tumor necrosis with hypothermia. Since then, cryoablation for low-risk estrogen receptor-positive/progesterone receptor-positive/human epidermal growth factor receptor 2-negative breast cancer ≤1.5 cm without resection has been shown to be safe and effective.^1,2^ Furthermore, we found cryoablation of the primary tumor and forgoing sentinel node biopsy offer an oncologically safe and feasible minimally invasive office-based procedure for patients with early-stage, low-risk breast cancer.^3^ Serendipitously, cryoablation, as opposed to resection, allows for retaining tumor-specific antigens in situ, promoting antitumor immune response. Cryoablation is also cost effective and is associated with excellent oncologic, physical, sexual, and cosmetic outcomes.^4^ Cryoablation therefore allows for ultimate de-escalation of surgery in select patients by decreasing treatment burden and improving patients’ quality of life.

## Future

As more breast cancer patients become aware of cryoablation for low-risk subtypes, they are seeking out physicians who provide the service as an alternative to surgery, especially the elderly. However, at present no standard of care has been established for breast cancer cryoablation, with only one publication defining practical guidelines.^5^ The hypothesis that cryoablation leads to enhanced antitumor immune response needs to be thoroughly investigated, particularly for high-risk immunogenic breast cancers. As follow-up data accumulate, the safety and selection criteria for cryoablation will need to be defined for appropriate adoption of cryoablation. This will also entail a clearer definition of which patients can safely avoid sentinel node biopsy. These guidelines will need to consider optimism bias by patients and surgeons to ensure safety.^6^ In summary, cryoablation without surgical resection is a promising, less invasive approach for low-risk breast cancer, with an increased interest in leveraging the procedure for high-risk, hard-to-treat subtypes.
